# Supplementary material for: Disease-causing mutations in subunits of OXPHOS complex I affect certain physical interactions
Source: Sci Rep. 2019 Jul 10;9:9987. doi: 10.1038/s41598-019-46446-8 (PMC6620328; doi:10.1038/s41598-019-46446-8)
Supplement: Supplementary file 1 — Supplementary figures merged [file 41598_2019_46446_MOESM1_ESM.pdf]

Disease-causing mutations in subunits of OXPHOS complex I affect their physical interactions – Supplementary material

Gilad Barshad[1], Nicol Zlotinkov-Poznianski[1], Lihi Gal[2], Maya Schuldiner[2] and Dan Mishmar[1*]

[1]Department of Life Sciences, Ben-Gurion University of the Negev, Beer-Sheva, Israel

[2]Department of Molecular Genetics, Weizmann Institute of Science, Rehovot, Israel

*Corresponding author:

Prof. Dan Mishmar

Department of Life Sciences

Ben-Gurion University of the Negev

Beer Sheva 8410501, Israel

Tel: +972-8-6461355

Fax: +972-8-6461356

Email: dmishmar@bgu.ac.il

Figure S1

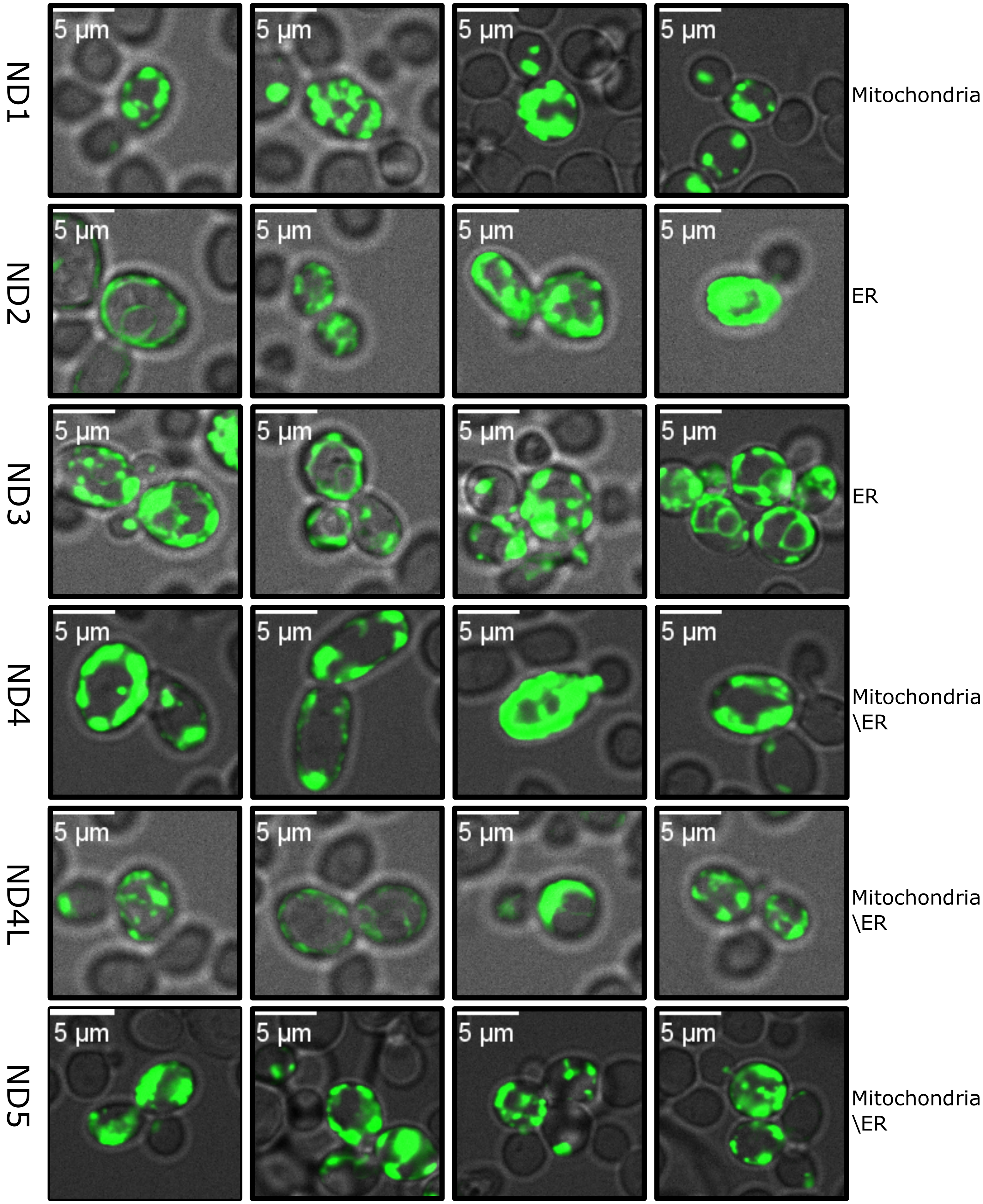
ND1
ND2
ND3
ND4
ND4L
ND5
5 µm
Mitochondria
ER
ER
Mitochondria \ER
Mitochondria \ER
Mitochondria \ER

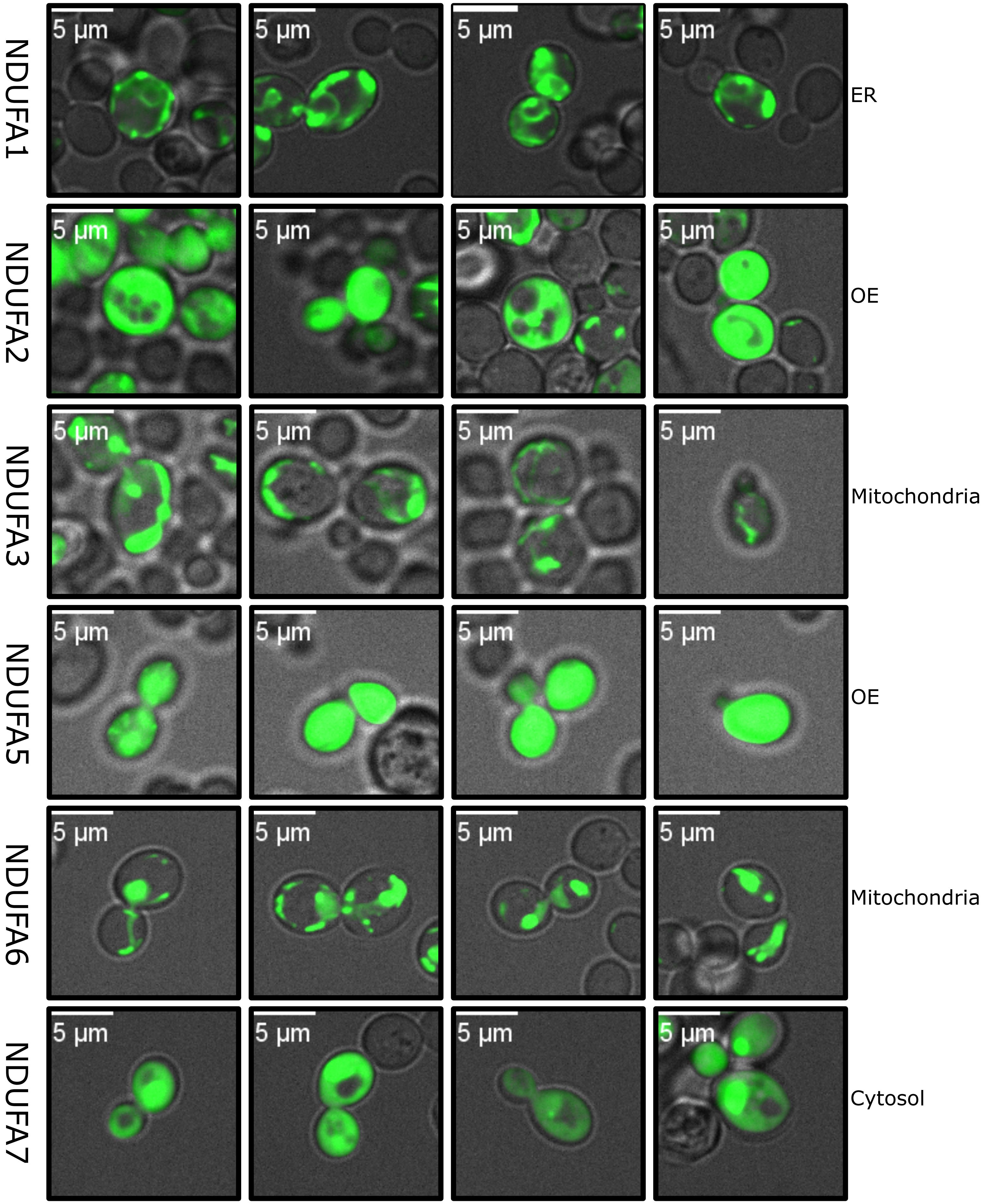

NDUFA1
NDUFA2
NDUFA3
NDUFA5
NDUFA6
NDUFA7
5 µm
ER
OE
Mitochondria
OE
Mitochondria
Cytosol

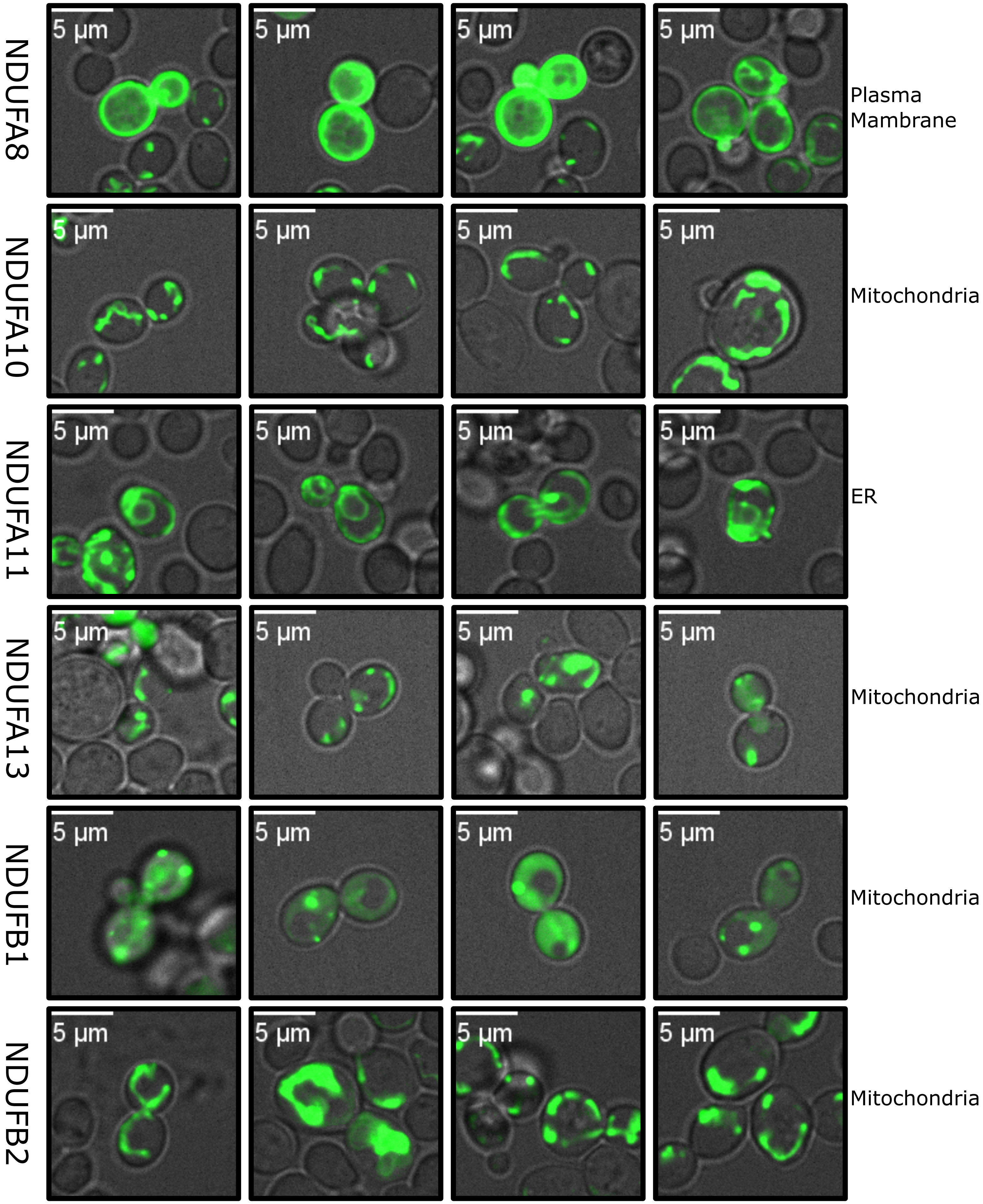
NDUFA8
NDUFA10
NDUFA11
NDUFA13
NDUFB1
NDUFB2
5 µm
Plasma Mambrane
Mitochondria
ER
Mitochondria
Mitochondria
Mitochondria

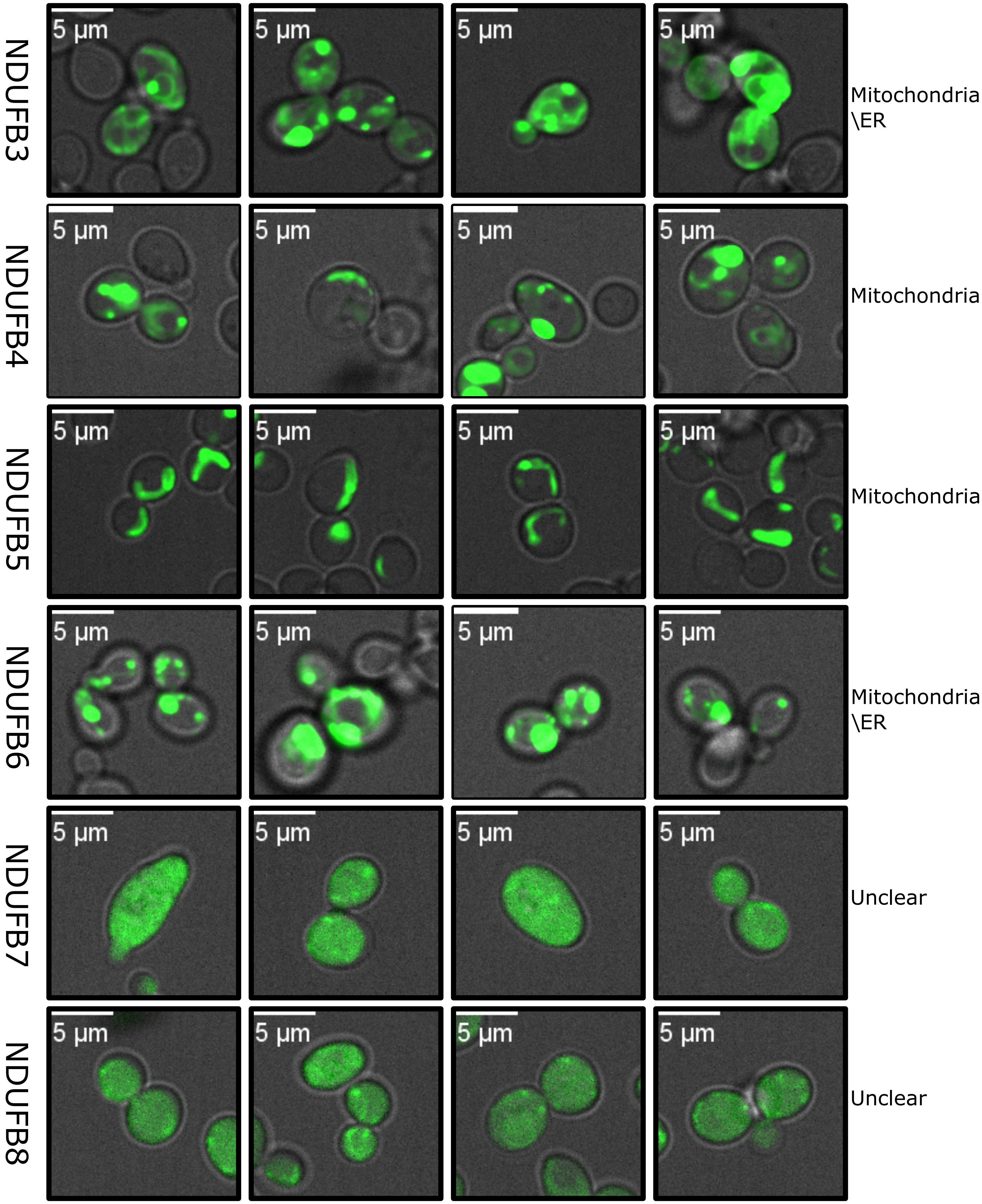
5 μm
NDUFB3
Mitochondria \ER
NDUFB4
Mitochondria
NDUFB5
Mitochondria
NDUFB6
Mitochondria \ER
NDUFB7
Unclear
NDUFB8
Unclear

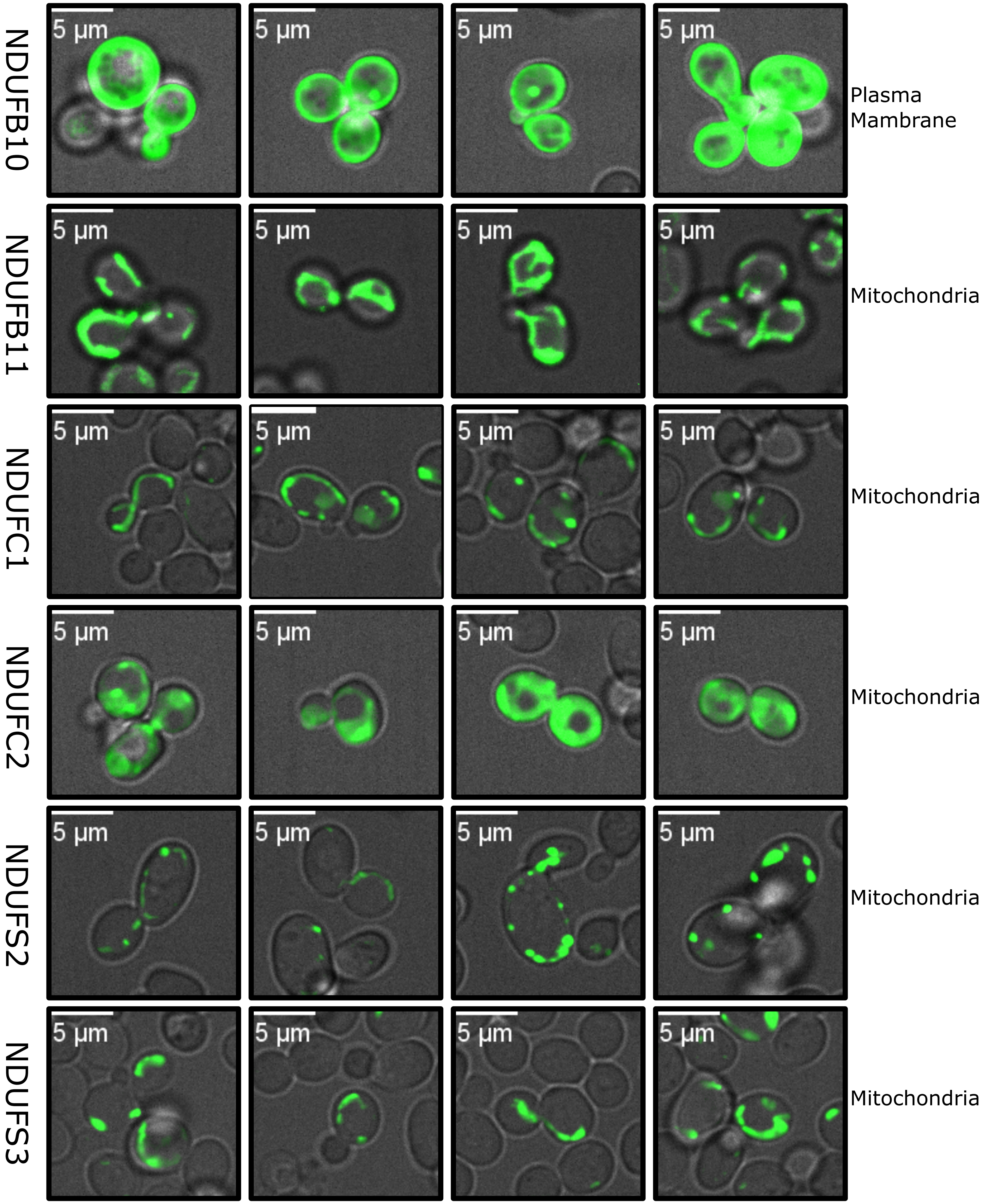
NDUFB10
NDUFB11
NDUFC1
NDUFC2
NDUFS2
NDUFS3
5 µm
Plasma Mambrane
Mitochondria
Mitochondria
Mitochondria
Mitochondria
Mitochondria

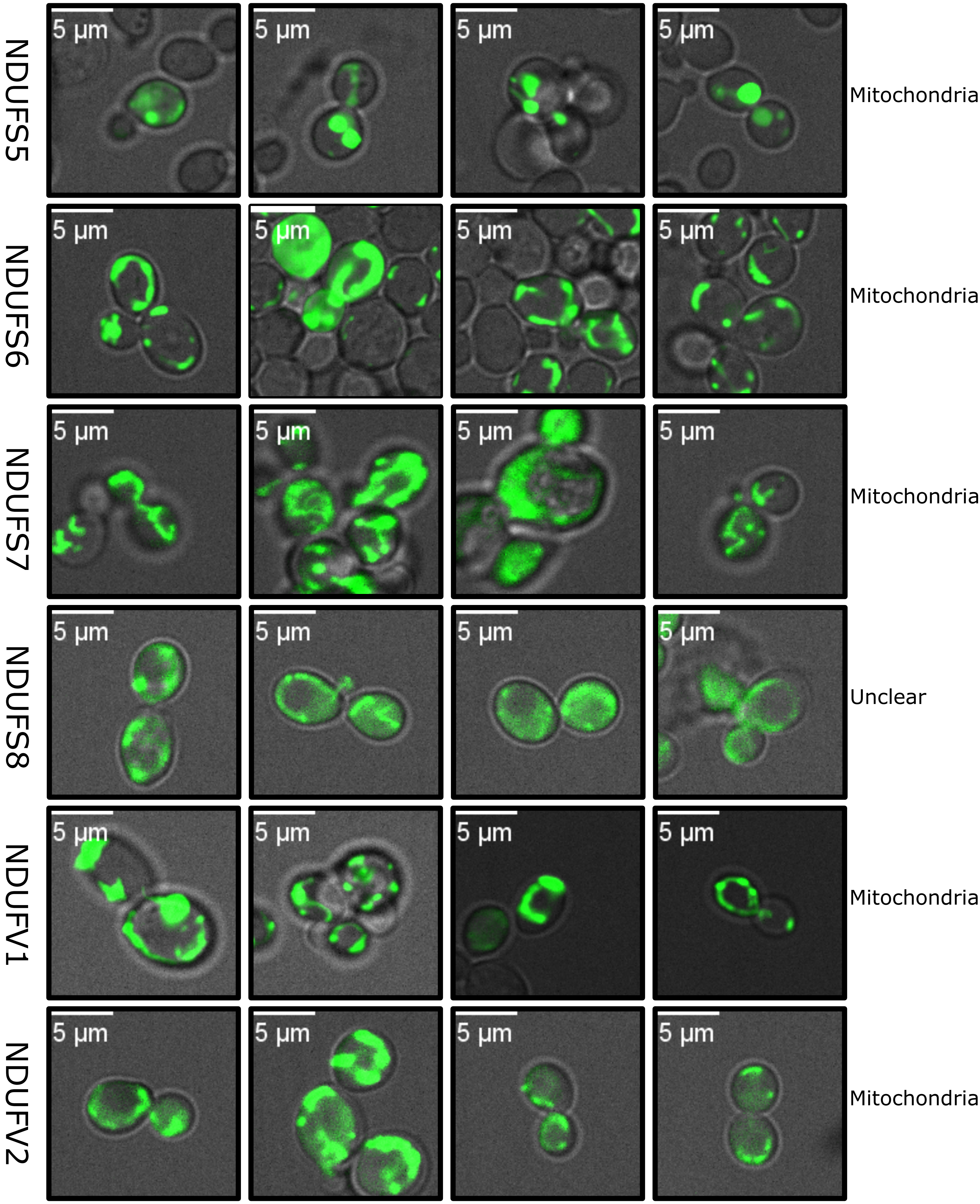

NDUFS5
NDUFS6
NDUFS7
NDUFS8
NDUFV1
NDUFV2
5 µm
Mitochondria
Mitochondria
Mitochondria
Unclear
Mitochondria
Mitochondria

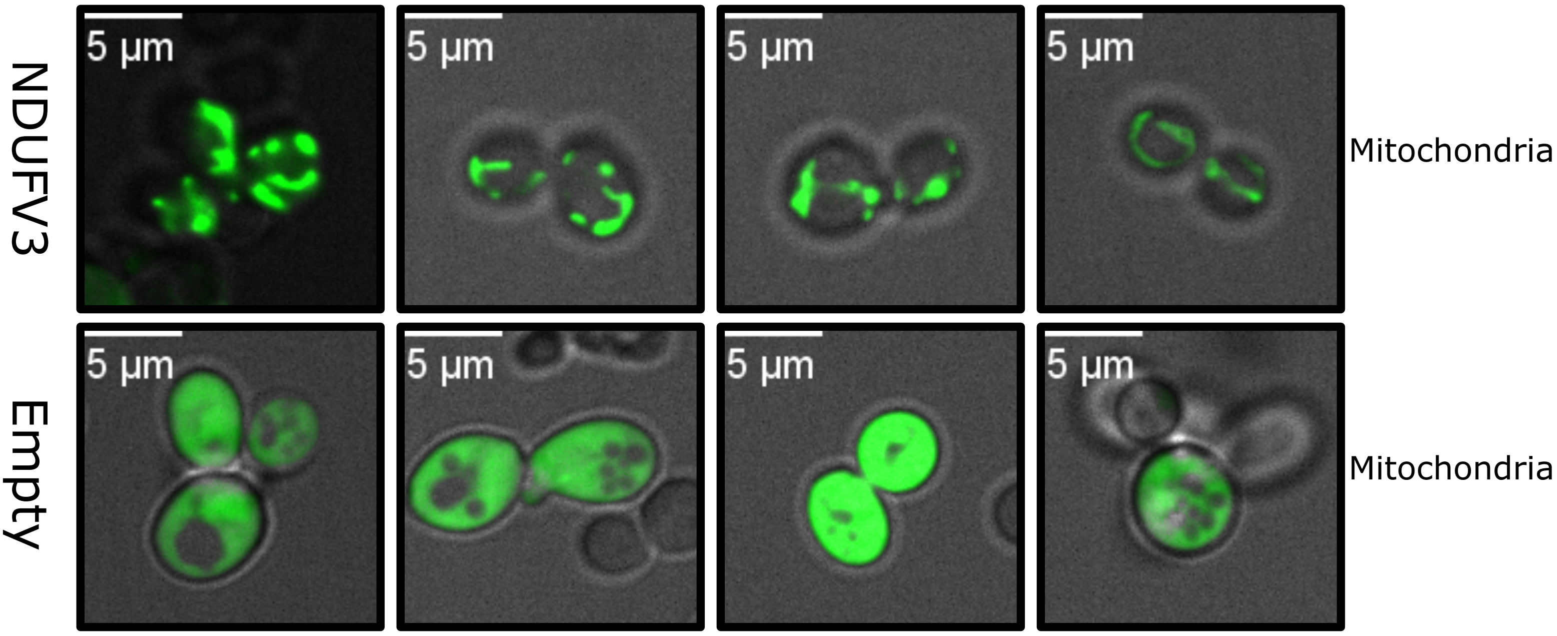

Figure S1. Localization of complex I subunits expressed within the haploid BY4742 yeast strain. The localization is based on YFP signal. OE - Over Expressed. Our constructs harbor a YFP fused to the F1,2 fragment of the mDHFR (in addition to the tested C1 subunit).

Figure S2

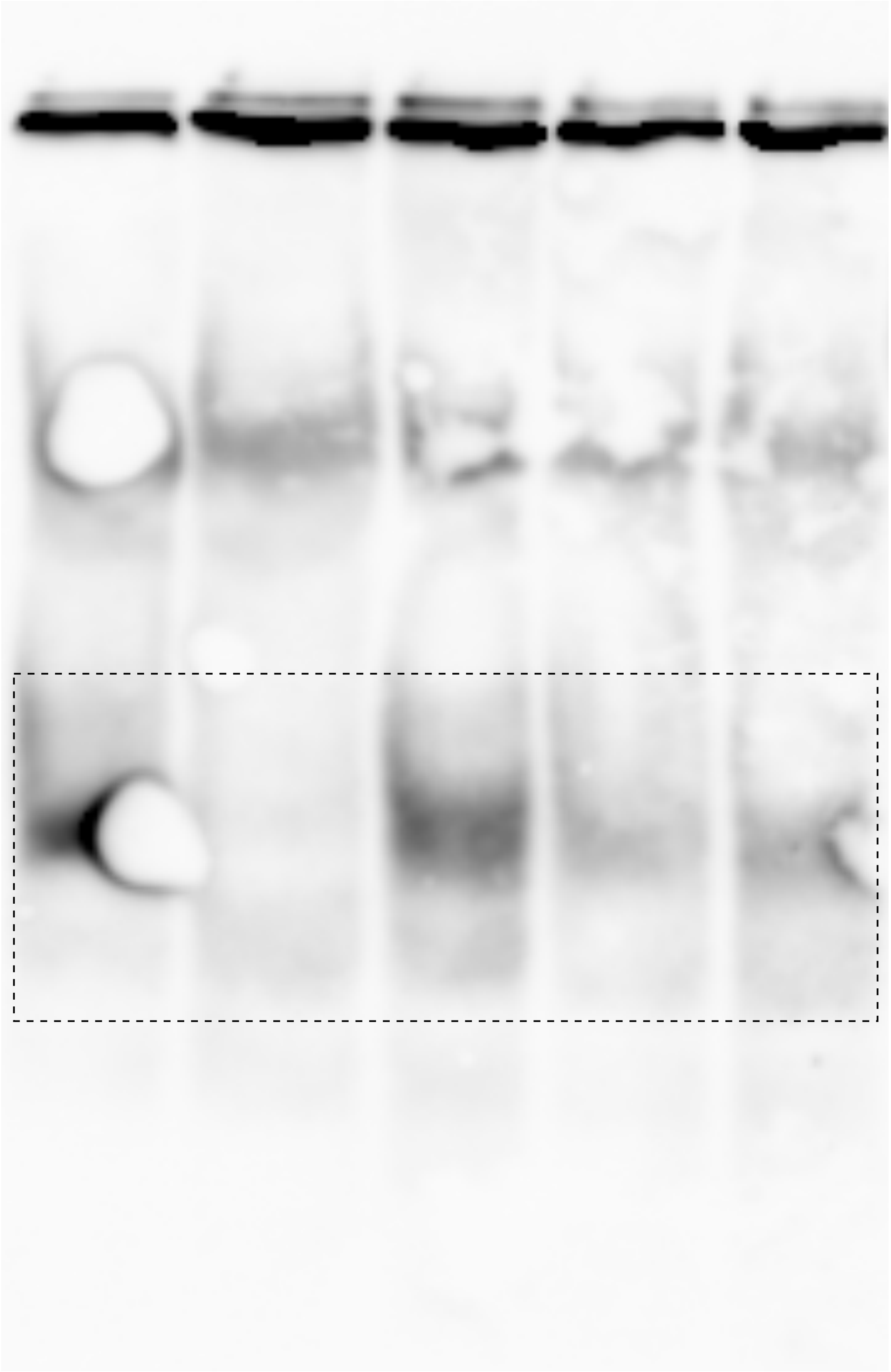

Figure S2. Full western blot complementary to Figure 3C. Dashed rectangle shown the area from which figure 3C was cropped.

Figure S3

A

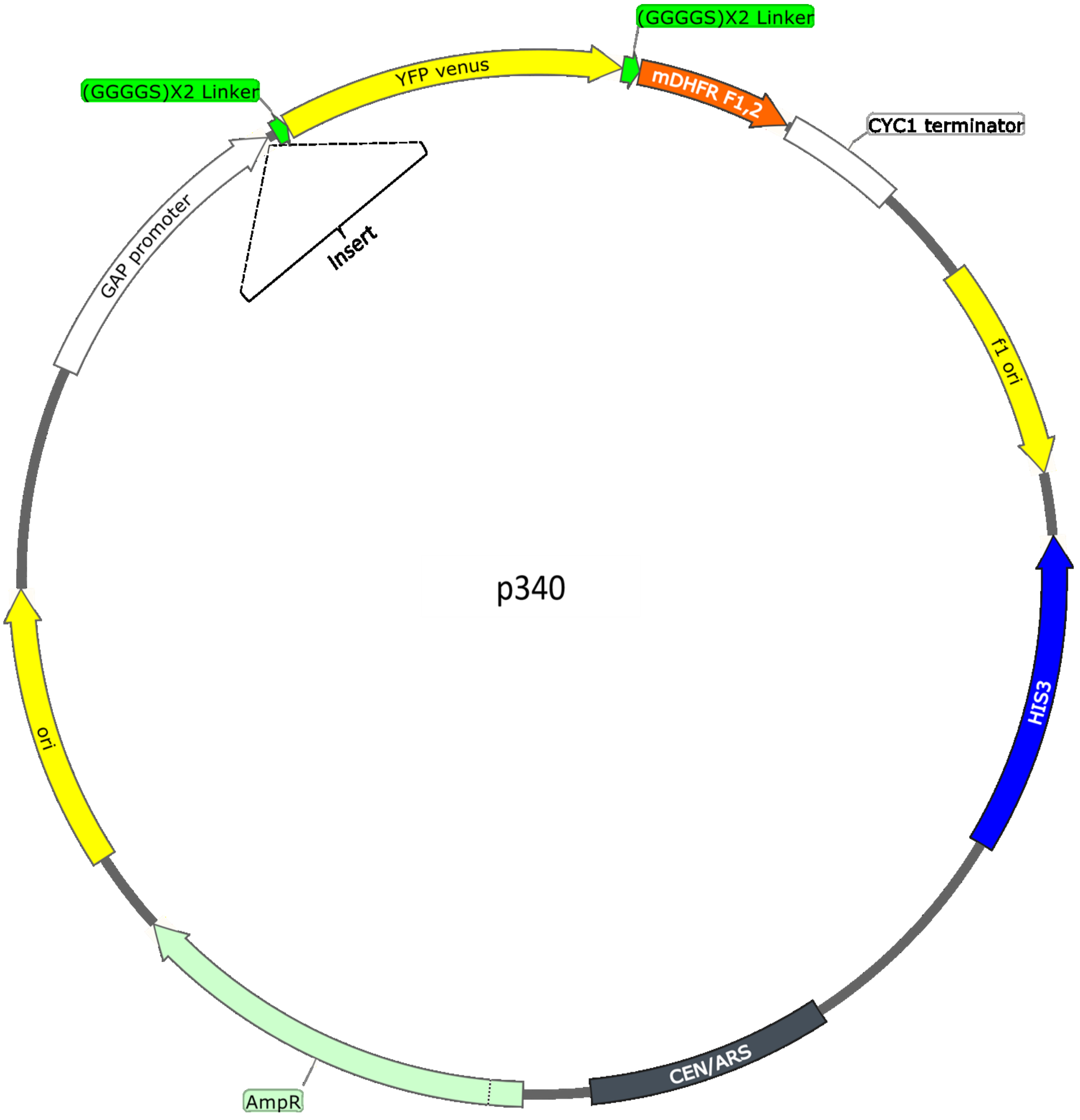

(GGGGS)X2 Linker
YFP venus
(GGGGS)X2 Linker
mDHFR F1,2
CYC1 terminator
GAP promoter
Insert
f1 ori
p340
HIS3
ori
AmpR
CEN/ARS

B

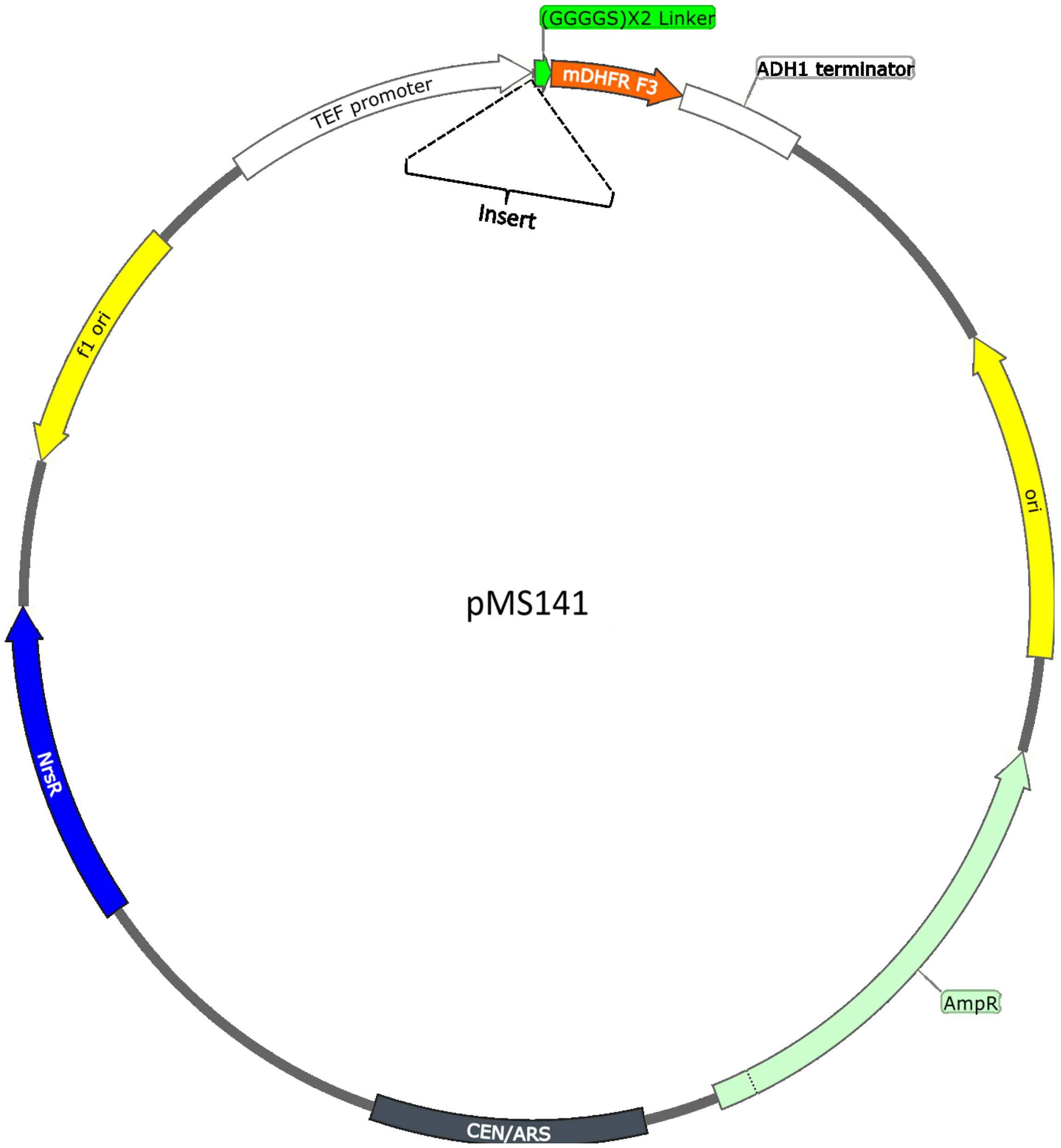

Figure S3. Maps of the constructs used in the split mDHFR PCA. Human CI subunit insertion sites in each plasmid are indicated.

Figure S4

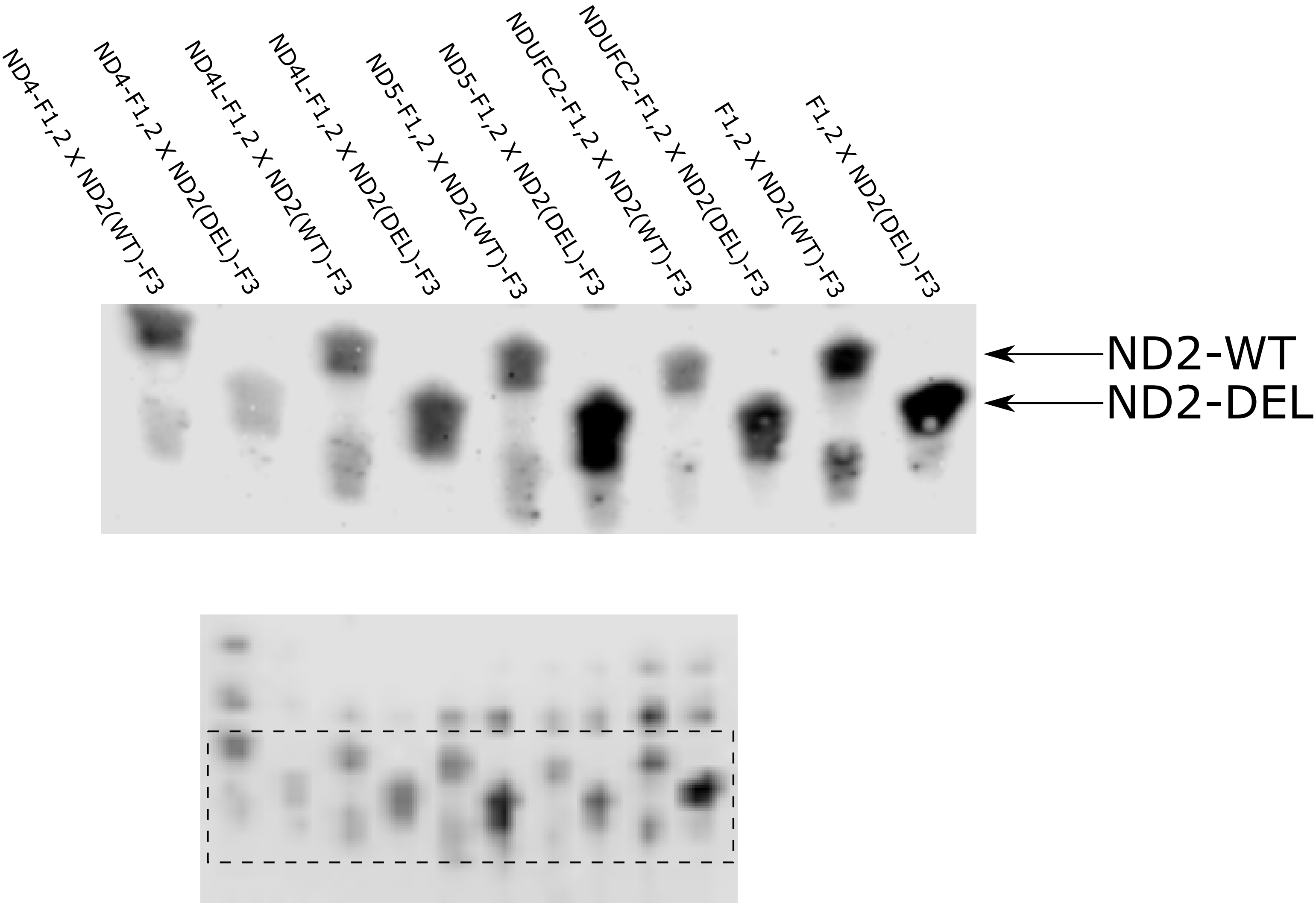

Figure S4. Validation of protein expression of the ND2 frameshift mutant and the wild type protein. Western blot representing three independent mating experiments of either wild type ND2 or the ND2 frameshift mutant with all four tested interactors. Bands representing the wild type and the frameshift mutant are indicated. Complementary full western blot is shown at the lower panel. Dashed rectangle represent the area from which the upper panel was cropped.
